# “*Don’t You Think It Is Violence Forcing Me to Have Sex While Not Happy?*” Women’s Conceptualization of Enjoyable Sex and Sexual Intimate Partner Violence in Mwanza, Tanzania

**DOI:** 10.3390/ijerph17217937

**Published:** 2020-10-29

**Authors:** Zaina Mchome, Gerry Mshana, Diana Aloyce, Esther Peter, Donati Malibwa, Annapoorna Dwarumpudi, Saidi Kapiga, Heidi Stöckl

**Affiliations:** 1 Mwanza Intervention Trials Unit, P.O. Box 11936 Mwanza, Tanzania; zsmchome@gmail.com (Z.M.); gerrymshana@hotmail.com (G.M.); diana.aloyce@mitu.or.tz (D.A.); esther.peter@mitu.or.tz (E.P.); donati.malibwa@mitu.or.tz (D.M.); Saidi.Kapiga@lshtm.ac.uk (S.K.); 2 National Institute for Medical Research, Mwanza Centre, P.O. Box 1462 Mwanza, Tanzania; 3 Gender Violence and Health Centre, Department of Global Health and Development, London School of Hygiene & Tropical Medicine, London WC1H 9SH, UK; anu.dwarumpudi@gmail.com; 4 Department of Infectious Disease Epidemiology, London School of Hygiene & Tropical Medicine, Keppel Street, London WC1E 7HT, UK

**Keywords:** intimate partner violence, sexual violence, qualitative interviews, Tanzania, sexual health

## Abstract

Intimate partner violence is a recognized public health and development issue that is consistently and comparatively measured through women’s experience of physical and/or sexual acts by their partner. While physical intimate partner violence is covered by a wide range of behaviors, sexual intimate partner violence (SIPV) is often only measured through attempted or completed forced sex, ignoring less obvious forms of sexual intimate partner violence. We explored women’s conceptualizations of SIPV by conducting in-depth interviews with 18 Tanzanian women. Using a thematic approach, we identified key features of women’s sexual intimate relationships and their perceptions of them. The women clearly defined acts of positive sexual relationships that occurred with mutual consent and seduction and SIPV that included acts of forced sex and sex under the threat of violence. They also identified several acts that were crossing the line, whereby a discrepancy of views existed whether they constituted SIPV, such as having sex when out of the mood, sex being the duty of the wife, sex during the menses, requests for anal sex, having sex to not lose the husband, husband refusing sex and husband having other partners. Women in this study felt violated by a far wider range of sexual acts in their relationships. Future studies need to improve the measurement of sexual intimate partner violence to allow the collection of encompassing, yet comparable, data on this harmful phenomenon.

## 1. Introduction

In recent years, there has been increased attention on understanding the multilayered causes of sexual violence and how it impacts women’s health, as well as on innovative prevention strategies to mitigate its varied consequences [1,2,3]. Sexual violence is commonly perpetrated by men known to the women, often their intimate partners [4]. A meta-analysis reports that in sub-Saharan Africa the pooled prevalence rate of sexual intimate partner violence is 18.8% [5]. In Tanzania, the 2015–2016 Demographic and Health Survey reports that 14% of women have experienced one or more acts of sexual violence, the most common being physically forced to have sexual intercourse by their spouse when they did not want to [6]. In Mwanza, Tanzania, 38% of women reported lifetime sexual intimate partner violence, and 17% reported it in the previous 12 months [7]. A study in Moshi, Tanzania, found that about 10.9% of women were sexually abused at first intercourse, and an additional 15.3% described their first sexual encounter as unwanted [8].

Sexual intimate partner violence is a global health problem that profoundly impacts women’s health. It is linked to gynecological problems, unplanned pregnancies, sexually transmitted infections, including HIV/AIDS, lack of sexual desire and loss of pleasure in sexual life as well as poor mental health and trauma [1,9,10,11,12,13]. Sexual violence at first intercourse in particular is associated with increased tolerance of violence later in life, suicidal attempts, increased risk of physical violence and having more sexual partners or partners with alcohol problems [8]. 

According to the World Health Organization (WHO), sexual violence is defined as “any sexual act, attempts to obtain a sexual act, or acts to traffic for sexual purposes, directed against a person using coercion, harassment or advances made by any person regardless of their relationship to the victim, in any setting, including but not limited to home and work” [14]. Sexual violence occurs in all societies, cultures and across all categories of difference—class, gender, race and ethnicity [15]. However, the meanings that different people attach to actions and behaviors—including those that constitute sexual violence—are best understood in a specific socio-cultural context. While there is an awareness of the importance of aligning the local knowledge and theoretical concepts in designing and implementing interventions that seek to address intimate partner violence, most programs in Tanzania and other low and middle-income countries mainly employ a conventional definition of sexual intimate partner violence as articulated by WHO.

Sexual intimate partner violence is a product of gendered relations, structures and practices of male power [9,16]. Sexual coercion and violence is prevalent in marriage, and most women endure abuse by partners quietly due to restrictive social and cultural norms [2,10,17] and stigma associated with separation or divorce [10]. Nonconsensual sex in marriage is part of the larger cultural landscape of many societies where sex in marriage is believed to be a man’s right [8,10,17]. This situation is exacerbated by religious interpretations which emphasize that women’s existence is to fulfill men’s sexual needs and to bear children [18]. This means that women in marriage are expected to be willing to have sex at any time [19]. 

Previous research on sexual violence in Tanzania and in other sub-Saharan African countries, has focused on community perceptions of intimate partner violence [20], prevalence of intimate partner violence against women [7,21,22] and its association with HIV risk behaviors [12,23,24,25] and women’s first sexual intercourse [8]. These studies, however, do not explore how women conceptualize sexual intimate partner violence. The current study aims to fill this gap, and to explore how women conceptualize positive sexual relationships.

Drawing on the cultural schemas theory [26], we analyze the women’s narratives of what they consider to be sexual violence. According to D’Andrade [27], a community’s cultural meaning of systems are composed of shared cultural schemas—i.e., knowledge structures—that allow people to identify objects and events. These schemas may include beliefs, perceptions, emotions, norms and values [26,28]. The schemas are context-specific, are powerful sources of knowledge and meanings and motivate people’s behavior and responses to familiar situations. Thus, how a certain behavior is conceptualized (e.g., sexual violence) depends on the cultural meaning systems of each community [26]. Some of the schemas (such as norms), however, are imposed and maintained through power differentials between social groups. These schemas are intended to normalize certain behaviors in the subordinate group. In this context, for example, such norms can normalize behaviors that constitute sexual violence. As structures, cultural schemas not only constrain individuals’ behaviors, but also make other forms of action possible [26]. For instance, while sex in marriage is culturally constructed as a man’s right, the negative sexual experience that women encounter in their relationships, may influence them to deconstruct this norm and refer to sex as consensual. In other words, although a woman is culturally expected to accept her husband’s sexual advances anytime, she may deny him sex when she has good reasons to do so (for example, when she does not feel like having sex).

## 2. Materials and Methods

### 2.1. Study Design and Methods

This paper draws on a qualitative component of the mixed-methods MAISHA longitudinal cohort study investigating the predictors and consequences of intimate partner violence in urban Mwanza. Participants of this longitudinal cohort study had previously participated in the control arm of the MAISHA cluster randomized controlled trial [29,30]. Between May and July 2019, we conducted 18 in-depth interviews to understand different forms of intimate partner violence from women’s own (*emic*) perspectives. The findings presented in this paper focus on sexual intimate partner violence. To assess the clarity and participants’ interpretation of the questions, we pilot-tested and adapted the topic guides of the interviews prior to the start of the actual fieldwork.

### 2.2. Study Participants and Recruitment

A total of 18 women aged 27 to 57 years were recruited from the longitudinal survey to participate in qualitative interviews which aimed to capture their understanding of different forms of intimate partner violence. (See Table 1 for participants’ information). The 18 women were purposively sampled from 85 women who reported changes in their experiences of sexual intimate partner violence between the baseline and endline survey of the MAISHA trial. The interviewers called the women to seek their consent to take part in the study and made appointments with those who agreed. The research team reviewed the data as the interviews continued, and stopped recruitment of new participants after the 18 interviews as it was found that no new information was coming from the interviews [31].

### 2.3. Data Collection

Two Tanzanian female research assistants, aged 26 and 27 years, were trained in conducting qualitative interviews. Topic guides with open-ended questions and probes were used to explore how women conceptualized sexual intimate partner violence. Given the sensitivity of the topic, opening questions were used to establish rapport and initiate the discussion, which included: Does it happen that your partner wants sex while you do not want to? What happens? Why? Interviewers also asked women specific questions to explore behaviors, actions and practices that constituted sexual violence. Those questions not only aimed to explore how women made sense of behaviors and practices that constitute sexual violence but also to hear the narratives and terms that they use to explain the phenomenon of sexual violence.

All interviews, which were audio recorded with the consent of participants, lasted between one and two hours, and were conducted in Swahili—the local language of the participants and researchers. Most of the participants were eager to share their perceptions and experiences, which contributed to a rich dataset. Detailed hand-written notes were made by the interviewers after every interview to document the key content of and any issues and emotions arising during the interviews. These observations were discussed during daily debriefing meetings to identify what issues needed to be explored further in subsequent interviews. Data collection continued by interviewing new participants until data saturation was reached [32]. The interviews were conducted at a time and place of participants’ choice, predominantly at their homes, or nearby hotel or restaurant that was sufficiently private to ensure that no other persons could overhear the conversations.

Ethical approval for this study was granted by the Ethics Committee of the London School of Hygiene and Tropical Medicine (Ref: 11918-3); and the National Health Research Ethics Committee (NatHREC) in Tanzania (NIMR/HQ/R.8a/Vol.1x12475). Prior to commencement of the study, the researchers also contacted local authorities for permission to conduct this study in their administrative areas. Full information was provided to participants verbally and written consent was obtained. Participants were reimbursed a total of 8000 Tanzanian shillings to help cover their transportation costs. All participants were provided with information on referral options for help and support and were assisted if they needed referral to specialized services. 

### 2.4. Data Analysis

All interview recordings were transcribed verbatim, and later translated into English. To assess the quality of the translation, a sample of the transcripts was translated back into English by a different translator. Inconsistencies were corrected before data analysis. All transcripts were analyzed in Swahili—the original language used to collect data for this study, and the local language of the researchers that led the data analysis. This enabled us to preserve linguistic authenticity [33], reduce a chance that the meanings of the participants accounts are lost, and increase the validity of the data that may be compromised in the translation process. The availability of English transcripts also enabled the English speaking researchers (including the 6th and last author) to explore the data set and contribute to the interpretation of the data, which increased rigor in analysis and enhanced richer interpretation of the findings. First, ZM read half of the transcripts line by line to identify initial inductive and deductive codes while cross-checking analytical concepts that had emerged during the fieldwork. The codes were then discussed and refined by ZM, GM and HS. Second, the patterns and relationships between the codes were identified and the main themes were synthesized—as described in Hennink, Hutter and Bailey [31]—which reflected sexual intimate partner violence. To ensure consistency, all transcripts were coded by two researchers using Nvivo 12 software, and then triple-checked by a third coder. Discrepancies were resolved through discussion by all authors. For the quotations, numbers rather than names are used to protect the participants’ identities.

## 3. Results

**Figure 1:**
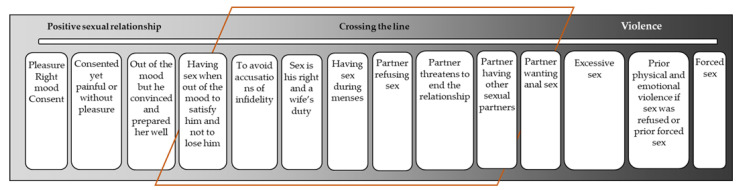
Women’s conceptualizations of sex and sexual violence.

In their accounts of what constitutes sexual violence, women in this study described three key themes as displayed in Figure 1: their conceptualization of positive sex, sexual behaviors that are crossing the line between acceptable forms of sexual behavior and sexual violence and clear forms of sexual violence. Although it was not the aim of this study to compare the participants based on ethnicity, we note that our analysis found no cultural differences in perspectives on sexual intimate partner violence between the ethnic groups represented in the study setting.

### 3.1. Women’s Conceptualization of Positive Sexual Relationship

Women’s accounts of positive sexual encounters clearly referred to what they perceived as an ideal sexual relationship and how enjoyable sex looks like. For most women, sex was associated with pleasure and seen as a sign for a strong relationship with their partner as long as it was based on mutual consent and the right mood.

Love is all about soothing one another, having consensus (on sex), and love should come from someone’s soul (*rohoni*). Don’t you think it is violence forcing me to have sex while not happy? You are supposed to soothe me, to entice me so that I agree, to make me look at you with romantic eyes…But if you force to have sex with me just because you are a man and you need it, that’s violence (*ukatili*).

(IDI-#04)

We reached an agreement and made love, but I felt the pain in my reproductive organs on the first day. I was so afraid when I felt the pain. But I felt okay.

(IDI-08)

In their descriptions of enjoyable sex, women often referred to the importance of being well prepared and being enticed with sexual foreplay, as quality foreplay would not only put them in the right mood for sex but also enable them to experience maximum satisfaction during sexual intercourse. A great foreplay was honored for not only making intercourse more enjoyable, but also preventing pain.

Mmm (showing sadness). I perceive having sex without being well prepared as abusive (*unyanyasaji*). He is supposed to prepare you first for that (act) instead of jumping over you in a rush. It’s not good. Preparations are prerequisite. You must do foreplay.

(IDI-#02)

Vindicating the majority of women’s views on quality preparations in relation to positive sex, one participant even indicated that a great foreplay by the partner before intercourse can make a woman enjoy sex even if she was initially not in the mood.

If the partner manages to convince you politely, and prepares you well before sex, you eventually feel like doing it to meet his needs. Even if you did (it) for him, you also enjoy it and never regret doing it.

(IDI-#16)

Women could still perceive sex as consensual and positive, even when they encountered physically painful experiences, most often reported when reflecting back on their first sexual intercourse. Many participants reported having negotiated with their partners before consenting to have sex for the first time, with some (men) approaching them directly, or using intermediaries who in most cases were friends. Thus, for most of the participants, their first sex was good as it was out of love and was consensual.

It was very good. Love is what influenced our decision to have sex. It is that love in him that influenced us to have sex.

(IDI-#05)

My mother used to sell *mbege* (local brew), so that boy used to come here as a customer. I then got used to him. During holidays, he would come and say that he wanted to take me out. One day, he took me out, and when we went there, it was the first time we made love, it was my first time and I didn’t even know how it was done. I didn’t know but he was old and experienced so he knew how. Truthfully, I felt so much pain for the first time, so much pain. Anhaa… after that, he didn’t do anything bad, I felt good, we kept on making stories then we went back home.

(IDI-#10)

Even if wanted though, some women did not have a positive recall of their first sexual intercourse, especially if it resulted in pregnancy. 

### 3.2. Sexual Behaviors that are Crossing the Line between Positive Sex and Sexual Violence

Women reflected on a range of sexual encounters they identified as problematic, with some but not all, of the women defining them as sexual intimate partner violence. 

#### 3.2.1. Having Sex Even When Not in the Mood 

Participants described that they often have a low sex-drive because of exhaustion from daily life packed with different duties, illness and stress. Sources of stress can be manifold, such as day-to-day difficulties in life, unresolved conflicts with their partner, trust or family issues or prior experiences of any form of abuse.

Sometimes you may have depression (*msongo wa mawazo*) due to life issues. Your head is full of stress. For sex to be enjoyable, you are supposed to be free of stress, and to be happy. You are supposed to be free, and to have nothing disturbing your head. That’s when you can enjoy that sex. Yes, sometimes things may confuse you to the extent that even when doing sex you don’t enjoy it at all.

(IDI-#14)

Another sexually violent behaviour is being raped. There are times that you do not want to have sex, but somebody forces you. He asks you, ‘will you give it to me or not’? You then end up fighting to the extent that you find it irritating and say ‘aah, let me give him (sex) so that I can sleep.’ That’s violence. When you force your fellow to have sex while she has requested you to excuse her as she is not okay is abusing her. That act (sex) is joyful and not just for the sake of doing it.

(IDI-07)

Participants commonly wished that their husbands would accept their reasons when they do not feel like having sex. Responding to the question about what she does not like from her partner in the current relationship, one participant, similar to many others, mentioned her husband’s tendency of forcing her to have sex against her will:

Honestly, what I don’t like from him is that sometimes you may not be in the mood (to do sex). As you know sometimes a man might be in need of sex but you (a woman) do not because we, women, do tough works. We spend all our day working. […] Therefore, it happens that at the time of sexual intercourse a woman is not in the mood […]. That means you are not ready for it when your partner wants to have sex with you. If your partner forces sex on you, that means he has abused you. It is violence (*ni ukatili*).

(IDI-#01)

Most participants described “having sex when not in mood” as a form of sexual violence, using terms as *ukatili* (cruelty), *unyanyasaji* (mistreatment) and *udhalilishaji* (humiliation). A few other participants went further to equate it with rape.

It (having sex out of mood) is not a good thing to begin with. He will hurt you, as you need to have the feelings so that the body prepares itself (to have sex). But if he just jumps on you by force, he will hurt you. That really is mistreatment, and it shouldn’t happen. Yes, why would he force you? That is equivalent to rape.

(IDI-#16)

When he forces you to have sex with him while you don’t want, that is harassment. He must be a rapist. Because he has forced you, you will regard him as a rapist. He takes you by force. Is he not a rapist?

(IDI-#02)

Preventing their husband’s infidelity surfaced as one of the common motivations behind women’s acceptance of unwanted sex in their relationships. There was a strong belief that men are sexually weak, with little ability for controlling their sexual urge. Thus, denying a husband sex would be a justifiable trigger for him having extra-marital sex. Based on that belief, women felt obligated to have sex even when they did not want to so they would not lose their partners to other women.

#### 3.2.2. Sex to Avoid Accusations of Infidelity 

Some women knew that their refusal to have sex with their husband might be viewed by their husband as an indication that they are already sexually satisfied by other men. Thus, to avoid being accused of infidelity, women tend to comply with their husband’s sexual needs even when they are not in the right mood.

He might even accuse you of infidelity because how could you refuse him otherwise? That’s where the problem is.

(IDI-#01)

Sometimes I don’t want to have sex but I just find myself accepting to give him what he wants. It is just because of his complaints saying “you have other men … this and that. You have had sex outside this and that”. You find yourself saying anyway, let me just give him what he wants.

(IDI-#14)

#### 3.2.3. Sex is His Right and a Wife’s Duty 

Albeit that the majority participants considered nonconsensual sex as violence, many were reported to abide with their husbands’ sexual desires, even when they did not want to. The women’s normalization of non-consensual sex was influenced by the cultural belief that sex is the right of a husband with his wife, thus denying him is violating the right he gained through marriage. While some women accept this and do not see it as violence, others challenge this belief.

It is his right as my husband but if you are not feeling anything it’s like he is really forcing you. But you tell yourself, let me do what he wants, what can I do?

(IDI-#02)

The obligation to satisfy their husband sexually was the most common reason for justifying women’s decision to have sex with their partner, even when not interested. This implies that women tend to value their husband’s sexual desires more than their own, and to accept unwanted sex to satisfy him.

At times when he forces me to make love, mm … sometimes you agree sometimes you refuse. In that case I do it to satisfy him. […] It is just normal.

(IDI-#05)

He usually gets mad at me when telling him that I don’t feel like having sex. After seeing that he does not understand me, and he becomes irritated, I decide to accept (to have sex). I just have to accept so to make him happy. But for me, I’m not satisfied as I did it unwillingly (*nimefanya tu kishingo upande*). Even so, I feel normal.

(IDI-#10)

#### 3.2.4. Having Sex during Menses

Almost all participants believed that having sex when in menstrual period is sexual violence, with Muslim participants depicting it as a forbidden behavior according to their religious doctrines. In this study, however, none of the women reported to have been forced to have sex during their menses.

That is a severe form of violence because he might bring you some infections. It’s disgusting and not proper at all when you have sex while on your menstrual period. It is problematic to a woman because of that dirtiness.

(IDI-#01)

Participants’ beliefs on sex as the husband’s right appeared to shape their conception of having sex when menstruating, so not all viewed it as sexual violence.

There are others who do not date out (have no mistresses), those who have only you. (If in period) I will explain to him that it is not good to have sex during the menstruation period, as he will get infections, especially the hydrocele disease (*busha*). But if you warn him and he refuses, you could clean yourself and let him in since you are his wife. And he shouldn’t complain if he gets infected. But I don’t think it is violence since he is asking for his right.

(IDI-#04)

#### 3.2.5. Partner Intentionally Refusing Her Sex

Participants framed sex as the core of any marital union, and a central motivation to get married. Sex was also perceived as the right of each partner, and each partner’s comfort to the other. In line with their conceptualization of sex, they considered a partner’s refusal of sex without good or justifiable reasons, including being turned on but left unattended, as a form of sexual abuse/violence (*unyanyasaji/ukatili*). Women were concerned with this behavior as in contrast to men, they could not decide to have extra-marital affairs to fulfill their sexual needs, as that would label them as deviant and promiscuous.

By doing that (denying sex), he would be not doing you justice (*atakuwa hajakutendea haki*), that’s cruelty (*ukatili*) or abuse (*unyanyasaji*). You are his wife, right? He didn’t marry you to cook or wash dishes. […] The central role of a wife is to give her husband comfort. Thus, when he refuses your comfort, it means he has abused you. It is abuse, because why would he refuse? And if he refuses, he should give reasons why.

(IDI-#01)

Participants mentioned circumstances that would make a man’s refusal of sex with his wife as not violent, including when the refusal happens rarely, or with justifiable reasons including being tired and the inability to perform sex.

It’s okay when it happens one time or once in a while. But when it becomes too much…Even if it were you … you would ask yourself and say “this person might have other sexual partners … why does he just sleep right away when he comes home?” or ‘why does he not have time for me?’ You will put a question mark there.

(IDI-#02)

#### 3.2.6. Being Threatened by the Partner of Ending the Relationship When Refusing Sex

Some women described how declining to have sex would lead to their partner threatening to leave them and engage in relationships with other women.

Threatening to leave you if you won’t have sex with him is abuse because why should he threaten to leave me, or do something? … A man can tell you that you don’t want to have sex with me; it is better if I look for another woman who will provide me with whenever I want, because you deny it to me sometimes, what for?

(IDI-#15)

It is violence if he scares me by saying he will leave me. I didn’t feel like doing it (sex). Let’s say he has upset me or I’m stressed, and you force me and scare me by saying you will leave me. I must do it so that you don’t leave me, I will do it but that will be violence.

(IDI-#11)

If he scares to leave me, its violence, why would you threaten to leave me? It’s violence because I didn’t feel like doing it. You may have upset me or I’m stressed, and you force me and scare to leave me, I must do it so that you don’t leave me. I will do it but that will be violence.

(IDI-12)

#### 3.2.7. Partner Having Other Sexual Partners

Almost all participants viewed a partner’s extra-marital relations as constituting sexual violence towards them, with many women in the study having experienced it. Their reasons were based on the consequences for them, including depression, low self-esteem, feeling less interested in sex with their partner, sexual denial by their husband, lack of intimacy, hatred against husbands, in addition to being worried about losing their partner to other women and contracting HIV. While explaining how her husband’s extra-marital affairs affected the way she expressed her sexual feelings, one participant said:

Lying is a sin, I can’t say a lie. Currently, it is not the same urge I used to have, to say that I am in need of him when I see him. Maybe I am in need of having sex with him, maybe I prepare him. Honestly, I don’t have that urge, maybe in the past. I could turn to him, hug him or even play with his nipples. So, things like those or maybe you touch him here and there and he notices that this woman feels this way. Yes, but for now, that is no longer there.

(IDI-#15)

One participant shared how her husband’s multiple extra-marital relations caused her harm through sadness and depression.

I started being sick after I had continuous stress, when I was pregnant with my baby who was ended up being a stillbirth … I think the problem was high blood pressure. When I was pregnant, I used to receive calls from different places. I received calls from different women telling me that ‘your husband has a woman here, please come if you can’. Only to find that his concubines were among those who were calling me. So, the situation that I was in caused me to have high blood pressure.

(IDI-#15)

Upholding the idea of male dominance on sexuality, some women depicted men’s extramarital affairs as expected, believing that a real man must have more than one sexual partner. In addition to extra-marital relations, having another wife was perceived as a form of sexual violence, particularly by Christian women as it is against their religious norms. For Muslim participants, however, this was not a form of sexual violence as it is in line with the Islamic norms, unless the man marries another woman without notifying his wife.

I think it is sexual violence as per my Christian faith. But it is okay for Muslims. I find it to be violence because when he goes to another wife, it indicates that he does not have true love for you. When he leaves, you know exactly where he is going and what he is going to do. You certainly know that he is going to have sex with another woman. For me that’s so painful. I feel that he does not do me justice.

(IDI-#14)

#### 3.2.8. Partner Wanting or Demanding Anal Sex

The majority of participants perceived the partner’s asking for or demanding anal sex as an immoral and sexually violent act. However, none of the participants reported to have ever experienced forced anal sex in their relationships.

Some men may want to do it [sex] opposite to the normal way (*kinyume na maumbile*). That is violence. […]. That (anus) is not a proper place that we are given (by God) to use for sex.

(IDI-#06)

That is a big offense! It is violence. I even don’t know what will happen when he asks for it (anal sex). I really hate it. In the first place, if a person asks you for that (anal sex) he has already crossed the line (*amevuka mipaka*). It is big mistake.

(IDI-#02)

Cultural norms and religious teachings emerged as important sources of schemas about anal sex and seem to have informed the participants’ framing of it as despicable, immoral and against nature.

A man may ask for sex against the nature (*kinyume na maumbile*). God doesn’t allow sex against the nature (anal sex), and the religious books (scriptures) too prohibit it. So, when someone tells you he wants to have sex with you from the other side, you definitely refuse so that you do not go against God’s commandments. […] As for me, my partner has never asked me for anal sex as we are all Muslims, our religion forbids that. It’s better not to mess-up with God’s laws. God created that organ (anus) for a special purpose, and this (vagina) is created for another purpose according to God’s plans. God assigned each of the two organs its own unique use. Thus, you can’t force to eat through the nose while there is a mouth to eat with, that’s impossible!

(IDI-#18)

During interviews, one participant reported that her partner used to ask her for anal sex, but she has been refusing. Unlike the majority women, she did not perceive his partner’s behavior of asking for anal sex as violence, as he never forced her to do it.

It would be violence if he would have forced me. But that never happened, so I am not sure if it is violence.

(IDI-#03)

Participants explained that men’s desire to have anal sex is influenced by peer pressure and their curiosity to explore it. They also added that in most cases men start to explore anal sex with women other than their partners/wives, and eventually demand the same from their partners. Thus, when a woman refuses his partner’s/husbands’ need for anal sex, she may face a myriad of consequences including being forced to have anal sex, being beaten, denied sex, receiving a partner’s poor economic support and a partner’s threat that he would seek that service from other women.

### 3.3. Uncontested Forms of Sexual Intimate Partner Violence

There were a number of behaviors that participants agreed unanimously clearly constitute sexual intimate partner violence. 

#### 3.3.1. Excessive Sex by the Partner

Participants expressed their concerns about excessive demands for sex, including needing sex very frequently or wanting several rounds of sexual intercourse in a single encounter. For instance, one woman, who was married at the age of 16, explained that her husband was abusive because he wanted sex frequently.

I was still young. At that time, I was feeling the pain in my soul. It was like torture, it was hurtful, and I reached a point of regretting getting married, had I known things are like this … I became thin at the beginning as I would cook but not eat while worrying; ‘perhaps when he returns home today he would demand for sex again’. Sometimes I would plead with him on my knees begging ‘please let us not do sex today’, but he says, ‘I can’t stop it; this is for every day’. […] When you tell him that you are hurt after an intercourse, he tells you that ‘when you are hurt, you need to keep doing it, that’s how you can get healed’.

(IDI-#16)

We have a neighbor, in an adjacent house, who is married to a young man. That young man wants sex from his wife whenever he comes home! Even when the woman has got work to do...When the woman refuses, she is assaulted in public. You can see what is happening. The woman is beaten in public (if she refuses sex)…and the man wants sex in the afternoon…in the morning; when he comes home (in the evening) … it doesn’t take very long before he wants sex again … and the girl is beaten up if she refuses.

(IDI-#02)

Additionally, when asked of any behavior that she may regard as sexual violence or abusive, one woman pointed to her current husband’s tendency of needing several rounds of sex while she is tired:

He has gone one round and he has ejaculated (*amemaliza*). Then he is forcing another round while you are tired. If I say am tired you have to understand that I am tired. Especially those who spend longer time (*wenye masafa marefu*). There are other men who take hours on your chest…they would love to proceed with sex after spending forty-five to sixty minutes for one round. Yet, when he finishes, he wants me to immediately go for another round. Don’t you think that is violence? I really can’t! It is violence. A lot of people [women] are grumbling about that.

(IDI-#04)

#### 3.3.2. Prior or Co-Occurrence of Physical and Emotional Violence 

The wife’s refusal of sex with her husband emerged as the common source of disagreements and conflicts between couples, and in most cases resulted in emotional or physical violence by their partner including being insulted or being bitten. While justifying habitually succumbing to unwanted sex, many pointed to their wish to avoid problems with their partners, using a common Swahili idiom, *“Unafunika kombe mwanaharamu apite”* (literally meaning, cover the cup and let the bastard pass), which indicates normalization of what they perceive as sexual abuse because of prior abuse when refusing.

Some men are cruel; when you refuse sex, he slaps you. So, in order to avoid being beaten up, you accept to have sex unwillingly (*unakubali kwa shingo upande*). But in real sense, you have not consented. You do it even when it’s not what you want.

(IDI-#17)

You try to explain it to them but they don’t understand. Some people strangle and rape their partners. Others may rip open their partners’ undergarments. You see? Each one has her own difficulties. There are those who will continually avoid you, having been denied sex. It all depends.

(IDI-#01)

He would just tell you ‘Mama Juma, I really want this madly’. You tell him, ‘I am not ready’. That’s where the fight begins. He may tell you, ‘It is possible yesterday you had rough sex (extramarital sex) … You had it nasty’. He would get mad; he would be harsh when you try to talk to him. He would just answer me anyhow because you didn’t respond to his needs.

(IDI-#04)

#### 3.3.3. Forced Sex

While the majority of women depicted themselves as tolerant to forced sex by their partners, a few reported their attempts to resist sex when not in the mood. However, they described their partners as powerful and therefore they win:

I experience forced sex several times. It reached a point where we had conflicts but then he wins, he has always been defeating me.

(IDI-#09)

There was a day when I refused to have sex with him. We then had a fight as he said I probably had other men. He forced me even more. I then had to give in as I am afraid that he will beat me. You just have to agree. It was so painful.

(IDI-08)

Participants described how they are affected by being forced to have sex, including feeling pain during sex (which afterwards turns into feeling anger), being sad or unhappy and lacking peace and having reduced feelings to or less interest in sex with their husbands.

You will feel pain because when you are not aroused … it feels like someone is stabbing you with something hard. See? When the friction gets too high, you might get some bruises because you are not prepared for it as opposed to when you are sufficiently lubricated (where the friction is minimal). But most people do this. You just give in and then the deal is closed (Sounding sad). It’s because he wants it when you don’t.

(IDI-#01)

## 4. Discussion

In this study, we aimed to explore women’s conceptualization of sex and sexual intimate partner violence. Although participants integrated some elements of the WHO definition of sexual violence [14] in their local models, their conceptualization of sexual violence encompasses additional sets of behaviors by partners—that go beyond forced sex or attempted forced sex—and include sexually violent behaviors in a broader sense. These include a man threatening to leave a wife because she refused sex, wanting sex during her menses, having other sexual partners, wanting sex when she is not in the mood, intentionally refusing her sex, forcing her to have anal sex and demanding too frequently, as well as a woman having non-consensual sex because of previous experience of physical or emotional violence if she refuses. While most women did not consider these behaviors as acceptable, they were not unanimous in considering all of them to be sexual violence. 

Previous studies depict women’s and men’s perspectives on intimate partner violence, including sexual violence, to be hinged on their cultural expectations [17]. However, in this study, many of women’s constructs and meanings they attach to sexually violent behaviors diverged sharply from the cultural norms imposing and perpetuating sexual violence. Culturally, in Tanzania [8], as is in many sub-Saharan communities [17], sex in marriage is framed as a man’s right. Although women in this study were aware of this cultural expectation, many firmly expressed their discontent with nonconsensual sex in their relationships, with a few of them confirming to attempt to detest their partner’s sexual advances when they were out of mood or had other reasons. This implies that women’s constructs and meanings they attach to sexual violence are informed not only by their cultural meaning systems, but also by their conceptualization of enjoyable sex and individual experience with violence. 

Our study findings show contradictory and yet important views on intimate partner sexual violence. Women identify certain acts as intimate partner sexual violence but tolerate them in their sexual relationships. For instance, albeit their framing of nonconsensual sex as violence, participants admitted to abiding with their husbands’ sexual advances even when they were not in the mood. Similarly, notwithstanding the majority’s labeling of extra-marital sexual relations by their partners as acts of sexual violence, they appeared to uphold male dominance in sexuality. This indicates how deeply schemas on sexuality are embedded in culture and, as such, are regarded as normative. The finding on women’s acceptance of nonconsensual sex by their partners is also reported in previous studies elsewhere in East Africa [17], and it indicates that in patriarchal cultures, such as that of our study setting, the institution of marriage is permeated with power differentials between spouses, with the man in a dominant position and the woman in a subordinate position. Thus, any resistance of sex from the woman is perceived by men as an insult to their masculinity [34]. In Bourdieusian perspective, acts of non-consensual sex against women would constitute forms of symbolic violence. They are violent because they are imposed, maintained and justified through social norms of sexual respectability [35]. These actions too are symbolic because they impose certain meanings. From the perpetrators side, sexual violence is an expression of power and the justification of their dominance over women [14]. From the perspective of victims, as shown in our findings, sexual intimate partner violence is tolerated by some women because of different reasons including the feeling of being obliged to satisfy the husbands sexually, the belief that sex is the husband’s right, to avoid being accused of infidelity (that is, being sexually satisfied by other men), to protect one’s relationship as the husband could look for satisfaction in extra-marital affairs and to avoid different forms of violence and misunderstandings with the partner. 

Some women, however, resisted acts of nonconsensual sex. This indicates that, while social norms of respectability constrain these women by imposing sexual intimate partner violence, they also enable women to oppose them. Drawing on Giddens concept of duality of structure, the social norms of respectability (structure) and women’s opposition to them (agency) are in a duality [36]. 

Although women’s conceptualizations partly aligned with the global framework of sexual violence, it consisted of concepts that are not in the scope of the conventional definition of sexual intimate partner violence. Our study participants broadly conceptualized sexual violence beyond the sexual act or the attempt to obtain the sexual act. The partial congruency between the local and global concepts of sexual intimate partner violence is to be expected as concepts from different systems of thought rarely overlap perfectly [37]. Our findings highlight that the conceptualization of sexual violence by women in their socio-cultural context is broader than what is known. It points to the need of broadening our understanding of sexual violence by engaging those who experienced sexual intimate partner violence in its definition, and the need for updating the available framework to be more respectful of local models. As Hahn and Inhorn [38] have cautioned, ignoring the cultural context may lead recipients of interventions to reject the programmers’ advice, either because they do not understand it, or because they give it a relatively low priority. Thus, developing interventions against sexual intimate partner violence that build upon the women’s conceptual models could help intervention experts to make their advice feel more relevant and applicable to women in the community.

## 5. Strengths and Limitations of the Study

The current study has several strengths. Although intimate partner violence, particularly sexual violence is one of the most serious public health concerns affecting women, as far as we are aware, this is the first study in Tanzania to explore in-depth how women understand sexual violence in the context of intimate relationships. Additionally, analyzing the data in the original language by employing cultural schema theory [26] resulted in rich data. As this study was conducted in a small part of Mwanza city, and the number of participants was small, the results should be viewed as contextual, and may be applicable to the study setting and similar settings elsewhere. Nevertheless, having participants from different ethnic groups in our study sample, and the fact that some elements of symbolic violence related to sexual violence are also reported by other studies elsewhere in Tanzania [8] suggests that the findings are applicable to other parts of the country and remain an important way for women to understand and explain sexual intimate partner violence. The research assistants involved in collecting data for this study were younger than most of the participants, a marginal position in many African societies where deference to elders is a norm [39]. Notwithstanding, being women, masked the disadvantage of looking young to some of the participants as it brought them to the peer group of “adult women” able to enter into women’s stories with empathy.

## 6. Conclusions

This study adds to the growing body of evidence suggesting the importance of understanding sexual violence in a particular context. It points to the continuing need to integrate cultural models into the design and implementation of interventions aiming to mitigate sexual violence against women by intimate partners. The experience of women in this study illustrates that local understandings and the schemas regarding sexual violence may align to some extent with global conceptual models, but also indicates the necessity of a more in-depth understanding of cultural models to make programs about intimate partner violence relevant to women who experience it. Additionally, our findings indicate that women have a boarder definition of sexual violence. We suggest that the intervention programs and initiatives that focus on addressing women’s vulnerability to sexual intimate partner violence need to engage men by creating open discussions on what women consider as sexual intimate partner violence. Interventions of this nature will help men to develop a critical awareness of their own privilege and status within a context of unequal gendered structures and relationships which underlie sexual violence against women.

## Figures and Tables

**Table 1:** Characteristics of the study participants.

Participant ID	AGE	Marital Status	#Children <18	Level of Education	Occupation	Religion	Tribe
IDI-#01	43	Married	4	Secondary	Tailor	Christian	Ngoni
IDI-#02	45	Married	5	Primary	Farmer	Christian	Sukuma
IDI-#03	44	Married	2	Secondary	Farmer	Christian	Sukuma
IDI-#04	48	Widow	3	Primary	Entrepreneur	Muslim	Haya
IDI-#05	43	Married	1	Secondary	Unemployed	Muslim	Pare
IDI-#06	32	Divorced	2	Primary	Unemployed	Christian	Jita
IDI-#07	37	Married	3	Primary	Entrepreneur	Christian	Nyakyusa
IDI-#08	27	Single	1	Diploma *	Entrepreneur	Christian	Sukuma
IDI-#09	45	Married	1	Primary	Entrepreneur	Christian	Ngoni
IDI-#10	37	Divorced	4	Primary	Unemployed	Christian	Sukuma
IDI-#11	45	Married	none	Primary	Entrepreneur	Christian	Haya
IDI-#12	30	Married	2	Primary	Entrepreneur	Christian	Nyambo
IDI-#13	57	Married	1	Primary	Entrepreneur	Christian	Sukuma
IDI-#14	36	Divorce	3	Diploma *	Hotelier	Christian	Sukuma
IDI-#15	43	Married	2	Primary	Entrepreneur	Christian	Angaza
IDI-#16	41	Married	2	Primary	Entrepreneur	Muslim	Haya
IDI-#17	49	Married	1	Primary	Unemployed	Christian	Sukuma
IDI-#18	43	Married	2	Primary	Entrepreneur	Muslim	Sukuma

* A one to two-year program offered after secondary education focusing on a specific skill or field.

## References

[1] Jina R., Thomas L.S. (2013). Health consequences of sexual violence against women. Best Pract. Res. Clin. Obstet. Gynaecol..

[2] Puri M., Tamang J., Shah I. (2011). Suffering in silence: Consequences of sexual violence within marriage among young women in Nepal. BMC Public Health.

[3] DeGue S., Valle L.A., Holt M.K., Massetti G.M., Matjasko J.L., Tharp A.T. (2014). A systematic review of primary prevention strategies for sexual violence perpetration. Aggress. Violent Behav..

[4] Dartnall E., Jewkes R. (2013). Sexual violence against women: The scope of the problem. Best Pract. Res. Clin. Obstet. Gynaecol..

[5] Muluneh M.D., Stulz V., Francis L., Agho K. (2020). Gender Based Violence against Women in Sub-Saharan Africa: A Systematic Review and Meta-Analysis of Cross-Sectional Studies. Int. J. Environ. Res. Public Health.

[6] National Bureau of Statistics (NBS), ICF (2016). Tanzania Demographic and Health Survey and Malaria Indicator Survey (TDHS-MIS) 2015-16.

[7] Kapiga S., Harvey S., Muhammad A.K., Stöckl H., Mshana G., Hashim R., Hansen C., Lees S., Watts C. (2017). Prevalence of intimate partner violence and abuse and associated factors among women enrolled into a cluster randomised trial in northwestern Tanzania. BMC Public Health.

[8] Williams C.M., McCloskey L.A., Larsen U. (2008). Sexual violence at first intercourse against women in Moshi, northern Tanzania: Prevalence, risk factors, and consequences. Popul. Stud..

[9] Hien P.T.T. (2008). Sexual coercion within marriage in Quang Tri, Vietnam. Cult. Health Sex..

[10] Hussain R., Khan A. (2008). Women’s Perceptions and Experiences of Sexual Violence in Marital Relationships and Its Effect on Reproductive Health. Health Care Women Int..

[11] Kaye D.K., Mirembe F.M., Bantebya G., Johansson A., Ekstrom A.M. (2006). Domestic violence during pregnancy and risk of low birthweight and maternal complications: A prospective cohort study at Mulago Hospital, Uganda. Trop Med. Int. Health.

[12] Maman S., Yamanis T., Kouyoumdjian F., Watt M., Mbwambo J. (2010). Intimate Partner Violence and the Association With HIV Risk Behaviors Among Young Men in Dar es Salaam, Tanzania. J. Interpers. Violence.

[13] Campbell J.C. (2002). Health consequences of intimate partner violence. Lancet.

[14] WHO (2002). World Report on Violence and Health, Chapter 6.

[15] Kalra G., Bhugra D. (2013). Sexual violence against women: Understanding cross-cultural intersections. Indian J. Psychiatr..

[16] Abrahams N., Mathews S., Ramela P. (2006). Intersections of “sanitation, sexual coercion and girls” safety in schools. Trop. Med. Int. Health.

[17] Stern E., Heise L. (2019). Sexual coercion, consent and negotiation: Processes of change amongst couples participating in the Indashyikirwa programme in Rwanda. Cult. Health Sex..

[18] Moghadam V.M. (2004). Patriarchy in transition: Women and the changing family in the Middle East. J. Comp. Fam. Stud..

[19] Ganesh I.M. (1999). When sexuality is violence. Voices Chang. J. Commun. Dev..

[20] Laisser R.M., Nyström L., Lugina H.I., Emmelin M. (2011). Community perceptions of intimate partner violence—A qualitative study from urban Tanzania. BMC Womens Health.

[21] Mulawa M., Kajula L.J., Yamanis T.J., Balvanz P., Kilonzo M.N., Maman S. (2018). Perpetration and Victimization of Intimate Partner Violence Among Young Men and Women in Dar es Salaam, Tanzania. J. Interpers. Violence.

[22] Nyato D., Materu J., Kuringe E., Zoungrana J., Mjungu D., Lemwayi R., Manaji E., Mtenga B., Nnko S., Munisi G. (2019). Prevalence and correlates of partner violence among adolescent girls and young women: Evidence from baseline data of a cluster randomised trial in tanzania. PLoS ONE.

[23] Baumgartner J.N., Kaaya S., Karungula H., Kaale A., Headley J., Tolley E. (2015). Domestic Violence Among Adolescents in HIV Prevention Research in Tanzania: Participant Experiences and Measurement Issues. Matern. Child Health J..

[24] Lary H., Maman S., Katebalila M., McCauley A., Mbwambo J. (2004). Exploring the Association Between HIV and Violence: Young People’s Experiences with Infidelity, Violence and Forced Sex in Dar es Salaam, Tanzania. Int. Fam. Plan Perspect..

[25] Nyamhanga T.M., Frumence G. (2014). Gender context of sexual violence and HIV sexual risk behaviors among married women in Iringa Region, Tanzania. Glob. Health Action.

[26] D’Andrade R.G., Strauss C. (1992). Human Motives and Cultural Models.

[27] D’Andrade R.G., Shweder R.A., Levine R.A. (1984). Cultural meaning systems. Culture Theory: Essays on Mind, Self, and Emotion.

[28] Hill D.H., Cole M. (1995). Between Discourse and Schema: Reformulating a Cultural-Historical Approach to Culture and Mind. Anthropol. Educ. Q..

[29] Harvey S., Lees S., Mshana G., Pilger D., Hansen C., Kapiga S., Watts C. (2018). A cluster randomized controlled trial to assess the impact on intimate partner violence of a 10-session participatory gender training curriculum delivered to women taking part in a group-based microfinance loan scheme in Tanzania (MAISHA CRT01): Study pro. BMC Womens Health.

[30] Kapiga S., Harvey S., Mshana G., Hansen C.H., Mtolela G.J., Madaha F., Hashim R., Kapinga I., Mosha N., Abramsky T. (2019). A social empowerment intervention to prevent intimate partner violence against women in a microfinance scheme in Tanzania: Findings from the MAISHA cluster randomised controlled trial. Lancet Glob. Health.

[31] Hennink M., Hutter I., Bailey A. (2020). Qualitative Research Methods.

[32] Corbin J., Strauss A. (2012). Basics of Qualitative Research (3rd ed.): Techniques and Procedures for Developing Grounded Theory.

[33] Richards L., Morse J.M. (2012). README FIRST for a User’s Guide to Qualitative Methods.

[34] Elliott K. (2016). Caring Masculinities. Men Masc..

[35] Bourdieu P., Wacquant L.J.D. (1992). An Invitation to Reflexive Sociology.

[36] Giddens A.B. (1984). The Consititution of Society: Outline of the Theory of Structuration.

[37] Pool R. (1994). Dialogue and the Interpretation of Illness: Conversation in Cameroon Village.

[38] Hahn R.A., Inhorn M.C. (2009). Anthropology in Public Health. Bridging Differences in Culture and Society.

[39] Yacob-Haliso O. (2019). Intersectionalities and access in fieldwork in postconflict Liberia: Motherland, motherhood, and minefields. Afr. Aff..

